# Objective measurement methods for the evaluation of socket comfort in patients with transfemoral amputation: a systematic review

**DOI:** 10.3389/fbioe.2025.1576729

**Published:** 2025-05-30

**Authors:** Lisa Marie Tiesler, Melanie Edel, Fangxing Wang, Philipp Pieroh, Welf-Guntram Drossel, Alina Carabello, Daniel Zipplies, Christoph-Eckhard Heyde, Stefan Schleifenbaum

**Affiliations:** ^1^ ZESBO - Center for Research on Musculoskeletal Systems, Department of Orthopedic Surgery, Traumatology and Plastic Surgery, Leipzig University, Leipzig, Germany; ^2^ Department of Orthopedic Surgery, Traumatology and Plastic Surgery, Leipzig University, Leipzig, Germany; ^3^ Fraunhofer Institute for Machine Tools and Forming Technology IWU, Chemnitz, Germany; ^4^ Professorship Adaptronics and Lightweight Design in Production, Chemnitz University of Technology, Chemnitz, Germany; ^5^ Professorship Applied Thermodynamics, Chemnitz University of Technology, Chemnitz, Germany

**Keywords:** transfemoral, amputation, residual limb, prosthesis, socket, interface, comfort, measurement

## Abstract

**Objective:**

Achieving optimal fitting for the socket-limb interface in transfemoral amputees remains a significant challenge. This iterative fitting process largely relies on subjective feedback regarding the patient’s comfort and the expertise of the prosthetist. Consequently, this review aims to explore methods for identifying issues at the socket-limb interface through both objective and subjective measurement approaches.

**Methods:**

All articles available in MEDLINE and Web of Science up to May 2024 were screened and evaluated, with the authors conducting a quality assessment.

**Results:**

The socket design was the most frequently studied factor influencing the socket-limb interface (11/25), with investigations addressing challenges such as volume fluctuations (5/25), pressure and shear forces (4/25), femur pistoning (3/25), perspiration and ventilation (2/25), and prosthesis alignment (1/25). Objective measurement methods included gait analysis (6/25), mobility tests (7/25), radiological techniques (8/25), pressure sensors (5/25), and thermal sensors/imaging (2/25), as well as optical and metabolic assessments (3/25). Several studies (17/25) combined objective analyses with subjective questionnaires, such as the Socket Comfort Score (SCS) and Prosthesis Evaluation Questionnaire (PEQ), to evaluate comfort, satisfaction, and prosthetic preferences across varying socket designs. Individualized questionnaires addressing socket design preferences were also employed. Furthermore, a final clustered analysis was conducted to allow comparisons of approaches and tools used for examining similar issues. Despite methodological advancements, a lack of standardization in measurement approaches was evident.

**Conclusion:**

The findings of this systematic review highlight significant gaps in current methods for evaluating the socket-limb interface in transfemoral amputees. While both subjective questionnaires, such as the SCS and PEQ, and objective tools, including pressure sensors and motion analyses, offer valuable insights, neither approach alone is sufficient to comprehensively assess prosthetic fit and comfort. Methodological inconsistencies and the absence of standardized protocols further impede advancements in this field. This review underscores the need for a validated and standardized measurement method that combines subjective and objective approaches to enhance evaluation accuracy. Addressing these challenges will enable the development of reliable tools for assessing socket-limb interface quality, especially prosthetic fit and comfort, and drive progress in improving prosthetic functionality and patient outcomes.

**Systematic review registration:**

https://www.crd.york.ac.uk/PROSPERO/view/CRD42023405042, identifier, CRD42023405042

## 1 Introduction

Transfemoral amputation presents significant challenges for patients, particularly regarding mobility and quality of life. The primary approach to restoring function and independence in these individuals is the use of prosthetic devices. In 2019, 62,016 lower extremity amputations were recorded in Germany. Among these, transfemoral amputations had an incidence of 13.3 per 100,000 inhabitants, with the number of cases continuing to rise. Approximately half of all major lower limb amputations were attributed to peripheral arterial disease (Walter et al., 2022). Other indications for transfemoral amputations may include diabetes, trauma, infections, and tumors (Gottschalk, 1999). Patients with higher amputation levels often face increased difficulty in regaining mobility postoperatively, particularly older individuals with comorbidities such as diabetes and vascular disease, who constitute 60% of transfemoral amputees (Devinuwara et al., 2018). In contrast, individuals with trauma-related or non-vascular transfemoral amputations demonstrate higher rehabilitation success rates and better prosthetic fitting (Moore et al., 1989). Rehabilitation aims to restore mobility through the fitting of a prosthesis. This prosthetic intervention is crucial for maintaining independent living and quality of life. Generally, above-knee prostheses comprise the following components: foot, ankle, shank, knee, and socket. The socket is considered the most critical component, as it serves as the interface between the residual limb and the prosthetic device. Consequently, its design directly affects the comfort and functionality of the prosthesis (Kapp, 2000). Prosthetic fitting is a complex procedure because anatomical structures that typically do not bear weight in their natural state must adapt to this function within the socket (Beil et al., 2002). Moreover, the fitting of the prosthesis is largely dependent on the expertise of the prosthetist (Papaioannou et al., 2010). Adjustments to the socket are made through an iterative process based on the patients’ comfort, often assessed using questionnaires. Consequently, prosthetic fitting remains highly subjective, as no standardized, objective measurement methods are currently employed. This lack of consistency in prosthesis fitting (Ramírez-Patiño et al., 2015) results in 30%–57% of transfemoral amputees reporting dissatisfaction with the comfort of their prosthesis (Dillingham et al., 2001; Berke et al., 2010). Inadequate prosthesis fitting can lead to dermatological issues, such as ulcers and infections, which may potentially result in reduced usage or temporary non-use of the prosthesis (Lyon et al., 2000; Meulenbelt et al., 2009). To prevent such complications, prosthesis or socket properties must be tailored to the individual physiological characteristics of the residual limb. Based on this, evaluating socket designs necessitates a standardized, easily applicable objective measurement method validated against subjective feedback regarding comfort.

This systematic review evaluates whether socket fit and comfort can be assessed more reliably using objective measurement methods compared to subjective assessments via validated questionnaires. By emphasizing the integration and alignment of subjective and objective measurement methods, this review aims to advance the understanding of prosthesis-related challenges for transfemoral amputees and establish a foundation for future research.

## 2 Methods

The current systematic review was registered in the International Prospective Register of Systematic Reviews (PROSPERO; CRD42023405042) and conducted in accordance with the Preferred Reporting Items for Systematic Reviews and Meta-Analyses (PRISMA) guidelines (Page et al., 2021).

### 2.1 Search strategy

A comprehensive review of all literature published from 1980 to May 2024 was performed using the MEDLINE and Web of Science databases. The search was limited to publications in English to ensure accessibility and consistency. To frame the search process, the following clinical question was formulated using the Participant, Intervention, Comparison, and Outcome (PICO) framework: Can socket fit and comfort be assessed more reliably using objective measurement methods (O) in transfemoral amputees utilizing prostheses (P), whose socket-limb interface was technically analyzed (I), compared to relying solely on validated questionnaires (C)?

Validated questionnaires were selected as the comparison (C) method in this review because they serve as a benchmark for assessing the insights provided by established objective measurement methods. Their importance lies in their proven ability to address key factors like comfort, functionality, and patient satisfaction - critical dimensions in the evaluation of prosthetic socket fit for transfemoral amputees.

To identify relevant publication, a Boolean search string was developed containing the following combination of terms:

In MEDLINE: (transfemoral amput*) AND ((prosthe*) OR (socket) OR (interface) OR (residual limb)) NOT (osseointegrat*) NOT (THR) Filters: English.

In Web of Science: ALL = ((transfemoral amput*) AND ((prosthe*) OR (socket) OR (interface) OR (residual limb)) NOT (osseointegrat*) NOT (THR)) AND LA = (English).

**FIGURE 1:**
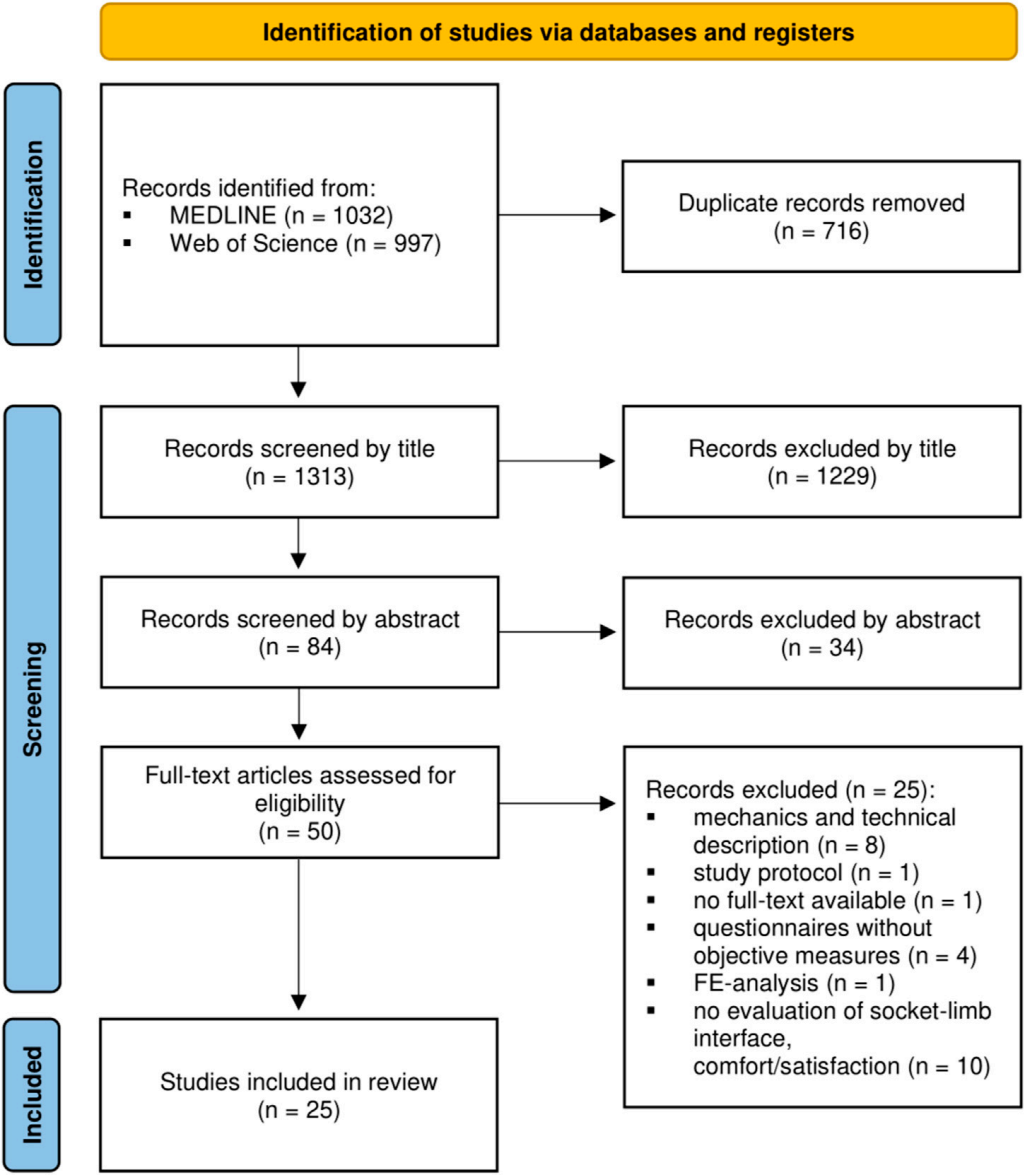
PRISMA flow diagram describing the search and study selection process.

In Web of Science, the search results were further filtered to limit the document types to “article”. After merging search results from both databases, duplicate entries were removed. Two reviewers (SS and LMT) independently assessed the search results using an iterative screening process (Figure 1, (Page et al., 2021)) based on predefined inclusion and exclusion criteria. Titles and abstracts were screened to identify potentially eligible studies, and full-text articles were analyzed in detail. Discrepancies between the reviewers were resolved through discussion to reach consensus.

### 2.2 Inclusion and exclusion criteria

Although the search string initially refined the search, additional restrictions ensured the inclusion of relevant studies. The inclusion criteria required that studies focus on unilateral transfemoral amputees utilizing prostheses and address the following aspects: (i) objective evaluations of different socket designs or the socket-limb interface; (ii) combined objective and subjective evaluations of different socket designs or the socket-limb interface; and (iii) challenges and influencing factors associated with socket-limb interface.

To provide a comprehensive overview, all objective and subjective measurement methods were included. This approach ensures that the analysis encompasses the full spectrum of tools used in studies, but regardless of their validation status.

Exclusion criteria ensured a clear focus and the removal of irrelevant or non-complementary studies. Thus, the exclusion criteria were defined as follows: (i) studies not published in English; (ii) studies without accessible full texts; (iii) studies exclusively investigating amputations of other anatomical regions (e.g., tibia or foot); (iv) studies on bone-anchored prostheses (users) or total hip replacements (THR); (v) research on the mechanics or technical descriptions of femoral prostheses; and (vi) studies employing the finite element method.

As not all criteria could be addressed by the search string, further adaptations using predefined restrictions were made. Consequently, mechanical analyses and technical descriptions were excluded as they do not represent patient-centered research. Furthermore, studies exclusively focusing on finite element methods were excluded, as these are considered computational approaches rather than direct objective measurement methods.

### 2.3 Quality assessment

The quality of the included studies was assessed using the framework by Brodie et al., 2022, which was adapted to address a similar research question. Their assessment methodology is based on established quality assessment tools (van Tulder et al., 1997; Verhagen et al., 1998) and additional reviews (van der Linde et al., 2004; Gholizadeh et al., 2014). Each criterion (Table 1 and Supplementary Material) was scored as “1” if its requirements were met and as “0” otherwise. Upon completing the evaluation process, a total score was calculated for each study. Furthermore, the level of evidence (LoE) for each publication was evaluated based on the simplified Oxford Centre for Evidence-Based Medicine criteria, using the following hierarchy (Brodie et al., 2022): (1) randomized trials, (2) nonrandomized trials, (3) cohort studies, (4) case series, and (5) expert opinions.

**TABLE 1:** Quality assessment criteria according to Brodie et al. (2022).

Category	Criteria	Description
Selection of patients	A1	Adequate description of inclusion and exclusion criteria
	A2	Functional homogeneity of included subjects (equal K-Level)
	A3	Comparability of group designs
	A4	Adequate randomization of group design or sequence of interventions
Intervention and assessment	B5	A detailed description of the experimental intervention
	B6	Cointerventions were avoided
	B7	Blinding of the assessor to the intervention
	B8	Adequate time of accommodation to adapt to the prosthesis
	B9	Adequate outcome measures
Statistical validity	C10	Sufficient reported dropouts (<20%)
	C11	Sample size exceeds 10:1 (participants completed protocol vs interventions)
	C12	Adequate data presentation

Note: Read the complete description of the listed criteria in the Supplementary Material.

### 2.4 Data extraction and analysis

Data extraction was performed using spreadsheet software (Microsoft Excel, version 2410, United States). Key information, including demographic details (e.g., age, gender), medical data (e.g., amputation level, prosthesis usage duration), and study designs, was summarized to facilitate comparative analysis. Additionally, the extracted data encompassed information on both objective measurement methods (e.g., pressure sensors, gait analysis) and subjective tools (e.g., validated questionnaires), as presented in Table 4. Discrepancies in data extraction were resolved through discussion and consensus to ensure accuracy and consistency.

Due to substantial heterogeneity in study designs, populations, and outcome measures, as well as the absence of randomized controlled trials, conducting a meta-analysis was not feasible. Instead, the findings were synthesized narratively, with a focus on highlighting methodological gaps and the potential to enhance prosthetic socket evaluation.

## 3 Results

The search string retrieved 1,032 publications from MEDLINE and 997 from Web of Science. After removing duplicates, a total of 1,313 articles remained for iterative screening. Following a thorough screening of titles, abstracts, and full-text articles, 25 studies were ultimately included in this review. Table 2 provides an overview of the demographic characteristics and content of these 25 selected studies.

**TABLE 2:** Summary of study characteristics and main facts.

Authors, Year of publication	Publication title	Level of amputation	Number of participants	Sex	Age in years	Etiology of amputation	Socket design	Time since amputation	Summary
Paternò et al. (2024)	Quantitative analysis of interface pressures in transfemoral prosthetic sockets	transfemoral	10	♂10	50 ± 9 (41–65)	trauma (N = 10)	IC, Sub-I, QL	24 ± 16 years (8–58 years)	Transfemoral amputees performed various locomotion tasks to investigate the pressure at the socket interface using their socket designs
Gnyawali et al. (2023)	Moisture mitigation using a vented liner and a vented socket system for individuals with transfemoral amputation	transfemoral	9	♂8 ♀1	46.5 ± 14.0	trauma (N = 8), infection (N = 1)	vented, non-vented	<1–15 years	When using a vented liner-socket system, the relative humidity and skin temperature of the residual limb can be significantly reduced. This does not cause significant changes in perceived stability, suspension, or comfort
Lee et al. (2023)	A pneumatically controlled prosthetic socket for transfemoral amputees	transfemoral	2	♂2	57 and 67	N/A	pneumatic	8 and 22 years	A pneumatically controlled prosthetic socket with two automatically adjusting air bladders was developed. The displacement between the socket and the residual limb was reduced by up to 33.4% due to air injection into the bladders
Cárdenas et al. (2022)	The effect of prosthetic alignment on the stump temperature and ground reaction forces during gait in transfemoral amputees	transfemoral	16	♂14 ♀2	37.8 ± 7.6 (25–53)	road accident (N = 10), gunshot (N = 3), cancer (N = 1), vascular (N = 1), workplace injury (N = 1)	QL	N/A	Misalignment of the femoral prosthesis can cause varying temperature distributions on the stump skin and different ground reaction force parameters
Diment et al. (2022)	Activity, socket fit, comfort and community participation in lower limb prosthesis users: A cambodian cohort study	transfemoral (N = 9), transtibial (N = 11)	20	♂15 ♀5	50 (24–60)	landmine (N = 11), traffic accident (N = 7), cancer (N = 1), infection (N = 1)	N/A	26 years (3-43 years)	Associations between perceived and measured activity levels including community engagement correlated with socket satisfaction in this cohort of Cambodian individuals with established lower limb amputations
Tang et al. (2022)	Analysis of lower limb prosthetic socket interface based on stress and motion measurements	transfemoral	1	♂1	29	N/A	supra-condylar suspension	>20 years	The pressure, shear and kinematic parameters of a transfemoral amputee were investigated over the course of 1 year. The greatest stresses were detected at the proximal-posterior and anterior-distal sensors in the socket. Pressure changes of up to 11% and changes in interface stresses of up to 40% were detected
Fatone et al. (2021)	Comparison of ischial containment and subischial sockets on comfort, function, quality of life, and satisfaction with device in persons with unilateral transfemoral amputation	transfemoral	25	♂19 ♀6	45.9 ± 13.7 (26–72)	trauma (N = 15), cancer (N = 7), vascular (N = 2), infection (N = 1)	IC, NU-FlexSIV	23.2 ± 15.8 years (2–53 years)	In persons with transfemoral amputation, the NU-FlexSIV socket was more comfortable and led to greater satisfaction than the IC socket, although this prosthesis did not improve functional tasks
Gale et al. (2021)	Residual limb shear strain during gait is correlated with patient reported outcomes for persons with transfemoral amputation	transfemoral	10	♂8 ♀2	51.9 ± 12.1 (24–63)	N/A	different IC and QL	N/A	Increased skin shear in the proximal and distal regions might decrease comfort and prosthetic use. The shear rate increased from the proximal to the distal parts of the residual limb
Kahle et al. (2021)	Effect of transfemoral prosthetic socket interface design on gait, balance, mobility, and preference	transfemoral (N = 11), knee disarticulation (N = 2)	13	♂10 ♀3	38.5 ± 11.0 (25–60)	trauma (N = 11), cancer (N = 1), congenital (N = 1)	IRC, DS, Sub-I	14.9 ± 12.7 years (2–34 years)	Each socket showed specific advantages and disadvantages, and the amputees had their preferred socket. Small differences in gait, mobility, and balance caused by different socket designs should be considered to determine the optimal socket interface
Maikos et al. (2021)	Effects of prosthetic socket design on residual femur motion using dynamic stereo x-ray	transfemoral	5	♂3 ♀2	48.8 ± 11.9 (33–61)	trauma (N = 4), cancer (N = 1)	IC, CRS	16.3 ± 14.3 years (3.1–39.0 years)	During gait, significantly less proximal-distal translation of the femur compared to the CRS socket was reported this caused no differences in comfort and usage
Paternò et al. (2021)	Residual limb volume fluctuations in transfemoral amputees	transfemoral	24	♂23 ♀1	53.3 ± 8.4 (39–65)	trauma (N = 24)	QL, IC, MA, Sub-I, CAT-CAM	21.1 ± 16.2 years (2–65 years)	Active use and removal of the prosthesis causes significant volume increases in the stump
Pellegrini et al. (2021)	The hybrid subischial socket for persons with transfemoral amputation: gait parameters and clinical assessment of a case series	transfemoral	3	♂3	18 and 35 and 32	trauma (N = 2), cancer (N = 1)	IC, HySS	10 months and 18 years and 7 months	The hybrid subischial socket improved passive and active hip range of motion, satisfaction, and speed in motion tests
Gale et al. (2020)	Motion of the residual femur within the socket during gait is associated with patient-reported problems in transfemoral amputees	transfemoral	10	♂8 ♀2	51.9 ± 11.4 (24–63)	N/A	N/A	N/A	Combined measurement with optical gait analyses and dynamic biplane radiography revealed a correlation between the pistoning of the residual femur and socket problems
Henao et al. (2020)	Influence of gait cycle loads on stress distribution at the residual limb/socket interface of transfemoral amputees: a finite element analysis	transfemoral	14	N/A	N/A	N/A	QL, NU-FlexSIV Semi-IC, Exo-modular	10.4 ± 8.7 years (1–42 years)	Comfort and discomfort were reported due to the prosthetic shaft of transfemoral amputees, which is associated with pressure-tolerant and sensitive areas on the residual limb
Kahle et al. (2020)	The effect of the transfemoral prosthetic socket interface designs on skeletal motion and socket comfort: a randomized clinical trail	transfemoral (N = 11), knee disarticulation (N = 2)	13	♂10 ♀3	38.5 ± 11 (25–60)	trauma (N = 11), cancer (N = 1), congenital (N = 1)	IRC, DS, Sub-I	14.9 ± 12.7 years (2–34 years)	Examinations of different socket designs for transfemoral amputees and individuals with knee disarticulation showed no significant influence on bone movement during walking or socket comfort
Aydın and Çağlar Okur (2018)	Effects of test socket on pain, prosthesis satisfaction, and functionality in patients with transfemoral and transtibial amputations	transfemoral (N = 25), transtibial (N = 63)	88	♂54 ♀34	40.3 ± 12.1 (18–70) (test socket), 38.9 ± 11.5 (18–68) (without test socket)	N/A	N/A	≥6 months	Preparing a test socket for transfemoral amputees decreases prosthesis-related problems such as pain and increases the time of use and function. A correct prosthetic socket fitting might offer 5–10 years of comfort and reliability
Brown et al. (2018)	Evaluation of NU-FlexSIV socket performance for military service members with transfemoral amputation	transfemoral	11	♂11	32.1 ± 4.0 (24–40)	N/A	IC, NU-FlexSIV	N/A	Using the NU-FlexSIV socket led to greater hip mobility in the sagittal plane compared to IC sockets. Other gait parameters were not affected. Various survey results on socket comfort and use were reported
Mitton et al. (2017)	Fluctuating residual limb volume accommodated with an adjustable, modular socket design: A novel case report	transfemoral	1	♀1	38	CRPS (N = 1)	adjustable-modular, conventional laminated thermoplastic	0–35 weeks	A conventional thermoplastic socket was replaced by an adjustable, modular socket during rehabilitation due to significant volume fluctuations, resulting in an immediate increase in wearing time
Kahle et al. (2016)	Comparative effectiveness of an adjustable transfemoral prosthetic interface accommodating volume fluctuation: Case study	transfemoral	1	♂1	24	sarcoma (N = 1)	IRC, adjustable transfemoral prosthetic interface	5 years	IRC shafts were compared with adjustable shafts in the event of volume fluctuations. In the volume loss condition, wearing an adjustable socket improved subjective and objective measures by up to 93% compared to standard care
Kahle and Highsmith (2014)	Transfemoral interfaces with vacuum assisted suspension comparison of gait, balance, and subjective analysis: Ischial containment versus brimless	transfemoral	10	♂8 ♀2	42.9 ± 14.7 (21–70)	trauma (N = 7), PVD (N = 2), sarcoma (N = 1)	IRC VAS, brimless IRC VAS	8.3 ± 10.1 years (0.8–26.0 years)	The brimless VAS interface demonstrated significant subjective improvements in prosthetic-related function and quality of life compared to the IRC interface
Kahle and Highsmith (2013)	Transfemoral sockets with vacuum-assisted suspension comparison of hip kinematics, socket position, contact pressure, and preference	transfemoral	9	♂7 ♀2	41.2 ± 14.5 (21–70)	trauma (N = 7), PVD (N = 1), sarcoma (N = 1)	IRC VAS, brimless IRC VAS	9.1 ± 10.3 years (0.8–26.0 years)	The brimless design caused significantly lower proximal medial pressure and proved to be more comfortable than the IRC design in the short term. Thus, it may be a clinically viable choice
Hoover et al. (2012)	The design and initial experimental validation of an active myoelectric transfemoral prosthesis	transfemoral	1	♂1	53	N/A	Myo-Socket (active myoelectric transfemoral prosthesis)	3 years	An active-knee prosthesis was developed for transfemoral amputees. It can modulate the knee’s flexion and extension using myoelectric signals from the residual limb
Klotz et al. (2011)	Influence of different types of sockets on the range of motion of the hip joint by the transfemoral amputee	transfemoral	4	♂4	51 ± 9 (42–63)	trauma (N = 3), vascular (N = 1)	QL, IC, IRC	>5 years	Wearing a prosthetic shaft decreases hip mobility compared to motion without a socket. The greatest restrictions were observed in adduction. The IRC socket allows the largest global range of motion
Hachisuka et al. (1999)	Subjective evaluations and objective measurements of ischial-ramal containment prosthesis	transfemoral	12	♂11 ♀1	46.2 ± 13.2 (IRC) 38.8 ± 14.2 (QL)	trauma (N = 10), atherosclerosis (N = 2)	IRC (N = 6), QL (N = 6)	7.7 ± 5.5 years (IRC) 3.9 ± 5.6 years (QL)	The IRC socket showed better functional results compared to the QL socket, but no metabolic advantages could be determined. The residual limb length ratio and lateral force may influence oxygen consumption
Lee et al. (1997)	Stump-socket interface pressure as an aid to socket design in prostheses for trans-femoral amputees - A preliminary study	transfemoral	2	N/A	47 and 58	N/A	QL, IC	≈30 and 40 years	The IC socket caused a more homogeneous pressure distribution than the QL socket. Both amputees preferred the IC socket.

The *Results* section is structured as follows to provide a clear presentation of the key findings, reflecting the complexity of this review, as initially addressed through the inclusion and exclusion criteria. This approach highlights the diversity of factors influencing the socket-limb interface and the varied methodologies used, which are categorized into distinct subsections to enable a systematic and focused presentation. Socket designs are examined in a separate subsection due to their essential role in shaping the socket-limb interface, which impacts both comfort and functionality. The final section presents a comparative analysis of the identified measurement methods, based on the PICO framework, to synthesize their contributions to the research question.

### 3.1 Quality assessment

Table 3 provides an overview of the total scores assigned to the included publications using the Brodie et al. (2022) framework. Scores ranged from 5 to 12 points, with one study achieving the maximum score of 12 points (Kahle et al., 2020). Three studies scored 10 points each (Klotz et al., 2011; Kahle and Highsmith, 2014; Kahle et al., 2021), while five studies achieved a score of 9 points (Fatone et al., 2021; Maikos et al., 2021; Cárdenas et al., 2022; Gnyawali et al., 2023; Lee et al., 2023). Seven studies scored 8 points (Lee et al., 1997; Hachisuka et al., 1999; Kahle and Highsmith, 2013; Aydın and Çağlar Okur, 2018; Paternò et al., 2021; 2024; Pellegrini et al., 2021). Five studies awarded 7 points (Hoover et al., 2012; Kahle et al., 2016; Mitton et al., 2017; Henao et al., 2020; Tang et al., 2022), while three studies received 6 points (Brown et al., 2018; Gale et al., 2020; 2021) and one study achieved 5 points (Diment et al., 2022).

**TABLE 3:** Quality and Level of Evidence (LoE) assessment.

Authors, Year of publication	Checklist questions												Score	LoE
	Selection of patients				Intervention and assessment					Statistical validity				
	A1	A2	A3	A4	B5	B6	B7	B8	B9	C10	C11	C12		
Paternò et al. (2024)	1	0	0	0	1	1	0	1	1	1	1	1	8/12	3
Gnyawali et al. (2023)	1	0	1	1	1	1	0	1	1	1	0	1	9/12	1
Lee et al. (2023)	1	1	1	0	1	1	0	1	1	1	0	1	9/12	4
Cárdenas et al. (2022)	1	0	0	0	1	1	1	1	1	1	1	1	9/12	3
Diment et al. (2022)	1	0	0	0	1	1	0	1	1	0	0	0	5/12	3
Tang et al. (2022)	1	0	0	0	1	1	0	1	1	1	0	1	7/12	4
Fatone et al. (2021)	1	0	0	1	1	1	1	1	1	0	1	1	9/12	1
Gale et al. (2021)	0	0	0	0	1	1	0	1	1	1	0	1	6/12	3
Kahle et al. (2021)	1	0	1	1	1	0	1	1	1	1	1	1	10/12	1
Maikos et al. (2021)	1	1	0	1	1	1	0	1	1	1	0	1	9/12	1
Paternò et al. (2021)	1	0	0	0	1	1	0	1	1	1	1	1	8/12	3
Pellegrini et al. (2021)	1	1	0	0	1	1	0	1	1	1	0	1	8/12	4
Gale et al. (2020)	0	0	0	0	1	1	0	1	1	1	0	1	6/12	3
Henao et al. (2020)	0	0	1	0	1	1	0	1	1	1	0	1	7/12	3
Kahle et al. (2020)	1	1	1	1	1	1	1	1	1	1	1	1	12/12	1
Aydın and Çağlar Okur (2018)	0	1	1	0	1	1	0	1	1	0	1	1	8/12	2
Brown et al. (2018)	1	1	0	0	1	1	0	1	1	0	0	0	6/12	4
Mitton et al. (2017)	1	0	0	0	1	1	0	1	1	1	0	1	7/12	4
Kahle et al. (2016)	1	0	0	0	1	1	1	1	1	0	0	1	7/12	4
Kahle and Highsmith (2014)	1	0	1	1	1	1	0	1	1	1	1	1	10/12	1
Kahle and Highsmith (2013)	1	0	1	1	1	1	0	1	1	0	0	1	8/12	1
Hoover et al. (2012)	1	0	0	0	1	1	0	1	1	1	0	1	7/12	4
Klotz et al. (2011)	1	1	1	1	1	1	0	1	1	1	0	1	10/12	1
Hachisuka et al. (1999)	1	1	0	0	1	1	0	1	1	1	0	1	8/12	2
Lee et al. (1997)	1	0	1	0	1	1	0	1	1	1	0	1	8/12	2

In terms of the level of evidence (LoE), summarized in Table 3, 8 out of 25 studies were classified as randomized trials with the highest LoE, 3 as nonrandomized trials, 7 as cohort studies, and another 7 as case series. No expert opinion studies (LoE 5) were included. The highest overall score (12/12, LoE 1) was attained by Kahle et al. (2020).

### 3.2 Socket designs

Depending on the research question, investigations used either pre-existing or newly fabricated prosthetic sockets. Most of the included studies focused on sockets specifically manufactured for research purposes (12/25) (Lee et al., 1997; 2023; Klotz et al., 2011; Hoover et al., 2012; Kahle and Highsmith, 2013; 2014; Kahle et al., 2016; 2020; 2021; Mitton et al., 2017; Fatone et al., 2021; Gnyawali et al., 2023). More specifically, the analysis identified eight studies that examined the standard prosthetic socket designs, known as ischial ramus containment (IRC, 6/25) (Klotz et al., 2011; Kahle and Highsmith, 2013; 2014; Kahle et al., 2016; 2020; 2021) or ischial containment socket (IC, 3/25) (Lee et al., 1997; Klotz et al., 2011; Fatone et al., 2021), in comparison to at least one other socket design addressing individual issues (Brodie et al., 2022). Nevertheless, Klotz et al. (2011) distinguished between the IRC and IC socket designs in their research. Quadrilateral sockets (QL) were assessed in two studies (2/25) (Lee et al., 1997; Klotz et al., 2011). Unlike the IC socket, the QL socket features a less elevated brim at the ischial tuberosity and lacks a bony lock in this area, but the body weight is still supported by this structure (Hachisuka et al., 1999). Two studies investigated dynamic sockets (DS) as an alternative to standard care (Kahle et al., 2020; 2021). This socket design incorporates lower trim lines and flexible interfaces to enhance comfort at the brim. Additionally, the DS features windows in the anterior and posterior socket walls, allowing muscle contractions. One study (1/25) compared the Northwestern University Flexible Subischial Vacuum (NU-FlexSIV) socket, characterized by the absence of an ischial seat, with the standard ischial care socket (Fatone et al., 2021). Omitting the ischial brim enabled this subischial socket design to achieve improvements in hip joint mobility and sitting comfort (Brown et al., 2018). As a consequence of amputation, volume fluctuations can occur throughout the day as well as over extended periods (intra- and inter-day) (Paternò et al., 2021). When such fluctuations make it challenging to fit a standard care prosthesis, adjustable sockets present a viable alternative for accommodating prosthetic fitting under difficult volume conditions (3/25) (Kahle et al., 2016; Mitton et al., 2017; Lee et al., 2023). Lee et al. (2023) developed a pneumatically controlled prosthetic socket equipped with two automatically adjusting air bladders to compensate for volume changes during gait. Another innovative approach is the myo-socket, which utilizes myoelectric signal detection from residual anatomical structures to compensate for limb loss (Hoover et al., 2012). Using this device, a transfemoral amputee is enabled to autonomously flex and extend the prosthetic knee. Addressing another common issue, Gnyawali et al. (2023) investigated a vented socket-liner combination in comparison to a non-vented device to optimize the socket-limb interface.

Alternatively, several studies compared participants’ existing sockets with newly manufactured ones (3/25) (Brown et al., 2018; Maikos et al., 2021; Pellegrini et al., 2021). All three studies compared the commonly used IC socket to the innovative designs, including the Compression/Release Stabilization (CRS) socket (Maikos et al., 2021), the Hybrid Subischial socket (HySS) (Pellegrini et al., 2021), and the NU-FlexSIV socket (Brown et al., 2018). The CRS socket is characterized by longitudinal depressions in its walls that facilitate compression and stabilization (Maikos et al., 2021). The HySS combines silicone with an external carbon fiber frame and employs suction suspension for enhanced fit and comfort (Pellegrini et al., 2021).

Additionally, seven of the included studies (7/25) examined the sockets habitually worn by the subjects (Hachisuka et al., 1999; Henao et al., 2020; Gale et al., 2021; Paternò et al., 2021; 2024; Diment et al., 2022; Tang et al., 2022). Hachisuka et al. (1999) selected participants based on the two socket designs under investigation (IRC and QL sockets). The habitual sockets used by these subjects are shown in Table 2. For three other publications, either no data regarding this classification were reported (Gale et al., 2020; Cárdenas et al., 2022) or the information was not considered relevant for this classification (Aydın and Çağlar Okur, 2018).

### 3.3 Challenges and influencing factors associated with the socket-limb interface

The review revealed that the studies addressed various challenges and factors influencing the socket-limb interface in unilateral transfemoral amputees, as summarized in Table 4. Eleven studies (11/25) focused on the optimization of socket shape and fit to ensure prolonged wearing time and enhanced functionality of the prosthesis (Lee et al., 1997; Hachisuka et al., 1999; Klotz et al., 2011; Kahle and Highsmith, 2013, 2014; Brown et al., 2018; Kahle et al., 2020, 2021; Fatone et al., 2021; Maikos et al., 2021; Pellegrini et al., 2021). Aydın and Çağlar Okur (2018) analyzed differences between an intervention group provided with test sockets as their initial prosthetic device post-amputation and a control group without test sockets. Since the preparation of test sockets is both financially demanding and time-consuming, the study evaluated their effects on functional and subjective outcomes during prosthesis use. Subjects using test sockets demonstrated significant improvements in daily walking distance, climbing stairs or slopes, reduced pain, and enhanced questionnaire scores compared to the control group.

**TABLE 4:** Summary of objective and subjective measurement methods.

Authors, Year of publication	Publication title	Challenges and influencing factors	Objective measurement	Subjective measurement
Paternò et al. (2024)	Quantitative analysis of interface pressures in transfemoral prosthetic sockets	pressure and shear rates	pressure sensors	none
Gnyawali et al. (2023)	Moisture mitigation using a vented liner and a vented socket system for individuals with transfemoral amputation	perspiration	relative humidity sensors, temperature sensors	CLASS, sweat score
Lee et al. (2023)	A pneumatically controlled prosthetic socket for transfemoral amputees	volume fluctuation	IMU, pressure sensors	none
Cárdenas et al. (2022)	The effect of prosthetic alignment on the stump temperature and ground reaction forces during gait in transfemoral amputees	prosthesis alignment	force platform, thermal camera	none
Diment et al. (2022)	Activity, socket fit, comfort and community participation in lower limb prosthesis users: A cambodian cohort study	volume fluctuation	accelerometer, 3D-scan system	individual questionnaire
Tang et al. (2022)	Analysis of lower limb prosthetic socket interface based on stress and motion measurements	pressure and shear rates	tri-axial pressure and shear sensors, motion capture	none
Fatone et al. (2021)	Comparison of ischial containment and subischial sockets on comfort, function, quality of life, and satisfaction with device in persons with unilateral transfemoral amputation	socket shape/design	mobility tests (5T-STST,4SST, AMP, T-test of agility)	SCS, OPUS
Gale et al. (2021)	Residual limb shear strain during gait is correlated with patient reported outcomes for persons with transfemoral amputation	pressure and shear rates	DBR, motion capture	Q-TFA
Kahle et al. (2021)	Effect of transfemoral prosthetic socket interface design on gait, balance, mobility, and preference	socket shape/design	pressure platform, mobility tests (2MWT, 4SST, 5T-STST, AMP), balance system	individual questionnaire (socket preference)
Maikos et al. (2021)	Effects of prosthetic socket design on residual femur motion using dynamic stereo x-ray	socket shape/design, pistoning	dynamic stereo X-ray, CT	combination of TAPES-Revised, PEQ, PPA
Paternò et al. (2021)	Residual limb volume fluctuations in transfemoral amputees	volume fluctuation	3D-scan system	none
Pellegrini et al. (2021)	The hybrid subischial socket for persons with transfemoral amputation: gait parameters and clinical assessment of a case series	socket shape/design	motion capture, force platform, mobility tests (6MWT, TUG), IMU, goniometer	SATPRO questionnaire
Gale et al. (2020)	Motion of the residual femur within the socket during gait is associated with patient-reported problems in transfemoral amputees	pistoning	motion capture, force platform, DBR, CT	Q-TFA
Henao et al. (2020)	Influence of gait cycle loads on stress distribution at the residual limb/socket interface of transfemoral amputees: a finite element analysis	pressure and shear rates	FE-analysis, CT	individual questionnaire
Kahle et al. (2020)	The effect of the transfemoral prosthetic socket interface designs on skeletal motion and socket comfort: a randomized clinical trail	socket shape/design, pistoning	X-ray	SCS
Aydın and Çağlar Okur (2018)	Effects of test socket on pain, prosthesis satisfaction, and functionality in patients with transfemoral and transtibial amputations	test socket	mobility tests (10-m walking on a flat surface, 10-step climbing up and down, 10-m walking up and down an 8% slope)	TAPES, BDI
Brown et al. (2018)	Evaluation of NU-FlexSIV socket performance for military service members with transfemoral amputation	socket shape/design	motion capture, mobility tests (T-test of agility, 4SST), obstacle course, force platform, fluoroscopy	SCS, combination of PAVET, PEQ, Q-TFA, TAPES
Mitton et al. (2017)	Fluctuating residual limb volume accommodated with an adjustable, modular socket design: A novel case report	volume fluctuation	wearing time	SCS
Kahle et al. (2016)	Comparative effectiveness of an adjustable transfemoral prosthetic interface accommodating volume fluctuation: Case study	volume fluctuation	mobility tests (2MWT, 4SST, AMP, L-test)	SIGAM mobility score, SCS, Pain Scale
Kahle and Highsmith (2014)	Transfemoral interfaces with vacuum assisted suspension comparison of gait, balance, and subjective analysis: Ischial containment versus brimless	socket shape/design	pressure platform, mobility tests (4SST), balance system	PEQ
Kahle and Highsmith (2013)	Transfemoral sockets with vacuum-assisted suspension comparison of hip kinematics, socket position, contact pressure, and preference	socket shape/design	X-ray, pressure sensors	individual questionnaire (socket preference)
Hoover et al. (2012)	The design and initial experimental validation of an active myoelectric transfemoral prosthesis	EMG, perspiration	EMG, walking speed	none
Klotz et al. (2011)	Influence of different types of sockets on the range of motion of the hip joint by the transfemoral amputee	socket shape/design	motion capture	none
Hachisuka et al. (1999)	Subjective evaluations and objective measurements of ischial-ramal containment prosthesis	socket shape/design	CT, X-ray, force platform, ECG	individual questionnaire
Lee et al. (1997)	Stump-socket interface pressure as an aid to socket design in prostheses for trans-femoral amputees - A preliminary study	socket shape/design	pressure sensors, force platform	none

Another critical issue frequently discussed is the fluctuation in residual limb volume and its effects on skin condition (5/25) (Kahle et al., 2016; Mitton et al., 2017; Paternò et al., 2021; Diment et al., 2022; Lee et al., 2023). Volume variations can affect prosthesis fit, altering pressure distribution and shear stress at the socket-limb interface (Paternò et al., 2018). Four studies (4/25) focused on the pressure or shear rates exerted on the stump by sockets used daily (Henao et al., 2020; Gale et al., 2021; Tang et al., 2022; Paternò et al., 2024). Tang et al. (2022) investigated the influence of walking speed on the pressure and shear rates at the socket-limb interface. These stresses are commonly associated with skin pathologies in transfemoral amputees. Furthermore, volume loss can lead to increased femur movement, referred to as “pistoning”, within the socket. Three authors specifically addressed this interface-related issue (Gale et al., 2020; Kahle et al., 2020; Maikos et al., 2021).

Another critical challenge when receiving a prosthesis is achieving the correct and optimal alignment of all components. In this context, Cárdenas et al. (2022) investigated deviations in prosthesis alignment. These deviations were induced through randomized translations and rotations of the optimal prosthesis setting. Incorrect prosthesis adjustments resulted in a shift of the center of gravity toward the sound side, creating asymmetry. This misalignment led to a shortened stance phase on the amputated side and increased loading on the sound limb. An innovative approach was pursued by Hoover et al. (2012), who used electromyographic (EMG) signals from the residual limb for self-determined control of a myoelectric prosthesis. In this device, the socket-limb interface not only posed challenges in terms of fit but also functioned as control unit for the prosthesis. By regulating knee flexion, the remaining thigh muscles were employed to compensate for the loss of control over the lower limb.

However, Hoover et al. (2012) reported additional challenges that affect the socket-limb interface. Excessive perspiration at the interface negatively impacted EMG signal detection, while volume fluctuations caused friction, resulting in movement artifacts. A further study investigated a solution approach that favors ventilation and the removal of perspiration through vented liner-socket systems (Gnyawali et al., 2023).

### 3.4 Objective measurement methods

Data collection methods can be classified broadly into dynamic gait analyses, mobility tests, and radiologic or (quasi-)static measurement techniques (Table 4).

Dynamic measurement methods include conventional gait analyses using optical, i.e., marker-based systems and/or inertial measurement units. Six of the included studies (6/25) analyzed various gait parameters, skin deformation, and hip joint range of motion using motion capture systems (Klotz et al., 2011; Brown et al., 2018; Gale et al., 2020; 2021; Pellegrini et al., 2021; Tang et al., 2022). Inertial measurement units (2/25) were mounted on the socket and liner, or at the pelvis and a lumbar vertebra, to detect socket displacement or measure the distance and duration of walking tasks (Pellegrini et al., 2021; Lee et al., 2023). Diment et al. (2022) employed accelerometers mounted on the prosthesis to record the activity levels of their study participants during prosthesis use.

Seven of the included studies (7/25) focused on defined mobility tasks for functional assessment of prostheses: 5-Times Sit-to-Stand Test (5T-STST, 2/25) (Fatone et al., 2021; Kahle et al., 2021), 4-Square Step Test (4SST, 5/25) (Kahle and Highsmith, 2014; Kahle et al., 2016; 2021; Brown et al., 2018; Fatone et al., 2021), Timed Up and Go Test (TUG, 1/25) (Pellegrini et al., 2021), T-Test of Agility (2/25) (Brown et al., 2018; Fatone et al., 2021), 2-Minute Walk Test (2MWT, 2/25) (Kahle et al., 2016; 2021), 6-Minute Walk Test (6MWT, 1/25) (Pellegrini et al., 2021), Amputee Mobility Predictor (AMP, 3/25) (Kahle et al., 2016; 2021; Fatone et al., 2021), and the L-Test (1/25) (Kahle et al., 2016). The investigation of the test sockets by Aydın and Çağlar Okur (2018) included measurements of the duration of daily use, walking distance, 10-m walking on a flat surface, climbing up and down 10 steps, as well as 10-m walking up and down an 8% slope. The 4SST was the most frequently performed mobility test (5/25) (Kahle and Highsmith, 2014; Kahle et al., 2016; 2021; Brown et al., 2018; Fatone et al., 2021). In the study by Brown et al. (2018), young transfemoral amputee military service members completed an obstacle course (1/25) in addition to two established mobility tests. Two further studies (2/25) used balance systems to assess the limits of patients’ stability (Kahle and Highsmith, 2014; Kahle et al., 2021).

Hachisuka et al. (1999) collected metabolic data (1/25) during walking with different prostheses by calculating the Physiological Cost Index (PCI) using electrocardiogram data and walking speed. The PCI was used to indirectly estimate oxygen consumption when wearing two different sockets: IRC and QL. The authors reported no significant difference in PCI between the two designs.

Five studies (5/25) employed pressure sensors, either inserted into the liner or integrated into the socket, to assess pressure and shear forces at the socket-limb interface (Lee et al., 1997; 2023; Kahle and Highsmith, 2013; Tang et al., 2022; Paternò et al., 2024). Lee et al. (2023) specifically utilized automatically adapting air bladders to measure pressure at the socket-limb interface. Additionally, pressure sensors (2/25) (Kahle and Highsmith, 2014; Kahle et al., 2021) and force platforms (6/25) (Lee et al., 1997; Hachisuka et al., 1999; Brown et al., 2018; Gale et al., 2020; Pellegrini et al., 2021; Cárdenas et al., 2022) were generally used for both dynamic and quasistatic measurements to detect ground reaction forces and loads on the extremities.

Another type of investigation were radiologic measurements, which were performed in eight studies (8/25) (Hachisuka et al., 1999; Kahle and Highsmith, 2013; Brown et al., 2018; Gale et al., 2020; 2021; Henao et al., 2020; Kahle et al., 2020; Maikos et al., 2021). These radiologic measurements can be categorized into dynamic and quasistatic setups. In two studies, dynamic biplane radiography (DBR, 2/25) was utilized to quantify skin deformation and residual femur motion within the socket (Gale et al., 2020; 2021). Maikos et al. (2021) employed dynamic stereo X-ray technology to detect residual femur motion within the socket. Computerized tomography (CT) was applied as an additional radiologic method (4/25) (Hachisuka et al., 1999; Gale et al., 2020; Henao et al., 2020; Maikos et al., 2021). CT scans were used to generate a 3D bone model for calculating femur motion on biplane radiographs (Gale et al., 2020; Maikos et al., 2021).

Fluoroscopic data enabled the determination of the relative motion between the residual femur and the socket. To analyze socket fit and the femur position within the socket, Hachisuka et al. (1999) performed CT scans of the residual limb in a donned condition. In their quasistatic simulation of gait events, Kahle and Highsmith (2013) investigated stance and swing phases of the amputated leg as well as bilateral loading on the lower extremities using X-rays and fluoroscopy. Fluoroscopy was also used to assess socket displacement during activity, both before and after applying load to the prosthesis (Brown et al., 2018). Either 0% or 100% quasistatic load was applied to the prosthesis. To simulate hip abduction during the middle stance phase, Hachisuka et al. (1999) captured X-ray images during single-leg stance. In another study by Kahle et al. (2020), coronal X-rays were obtained to analyze the position of bony structures within the socket (e.g., pistoning, lateral shifting, adduction) under conditions of unloading and full weight bearing. Pellegrini et al. (2021) determined the passive hip range of motion (ROM) using a goniometer.

Paternò et al. (2021) and Diment et al. (2022) examined the challenges posed by volume fluctuations in transfemoral amputees. To analyze these variations and the shape of the residual limb, both studies employed 3D scanning (2/25).

Using a thermal imaging camera, Cárdenas et al. (2022) conducted a quasistatic analysis of the residual limb to assess the impact of prosthetic misalignment. Their findings revealed a mean increase of at least 3.5% in the variation coefficient of temperature compared to the nominal alignment.

Another study used relative humidity and temperature sensors embedded in the socket to test vented liner-socket interfaces (Gnyawali et al., 2023). When using vented liner-socket interfaces, humidity levels were reduced by 30% relative to a non-vented system. Additionally, no significant rise in temperature was observed on the residual limb when comparing the two socket designs.

### 3.5 Questionnaires

17 of the 25 included studies (17/25) investigated patients’ subjective perception and satisfaction using questionnaires (Table 4) (Hachisuka et al., 1999; Kahle and Highsmith, 2013; 2014; Kahle et al., 2016; 2020; 2021; Mitton et al., 2017; Aydın and Çağlar Okur, 2018; Brown et al., 2018; Gale et al., 2020; 2021; Henao et al., 2020; Fatone et al., 2021; Maikos et al., 2021; Pellegrini et al., 2021; Diment et al., 2022; Gnyawali et al., 2023). These studies primarily focused on assessing comfort, quality of life, pain, and functionality related to amputation and prosthesis use. The Socket Comfort Score (SCS) was used in five studies (5/25) to assess socket comfort and identify the need for adjustments (Kahle et al., 2016; 2020; Mitton et al., 2017; Brown et al., 2018; Fatone et al., 2021).

Functional, social, and psychosocial aspects related to prosthesis use were analyzed using the following questionnaires: Trinity Amputation and Prosthetic Experience Scales (TAPES) (Aydın and Çağlar Okur, 2018), Prosthesis Evaluation Questionnaire (PEQ) (Kahle and Highsmith, 2014), Orthotic and Prosthetic Users’ Survey (OPUS) (Fatone et al., 2021), Questionnaire for Persons with a Transfemoral Amputation (Q-TFA) (Gale et al., 2020; 2021), Comprehensive Lower Limb Amputee Socket Survey (CLASS) (Gnyawali et al., 2023), and Satisfaction with Prosthesis (SATPRO) (Pellegrini et al., 2021).

Maikos et al. (2021) combined elements from existing validated questionnaires, such as the Prosthetic Profile of the Amputee (PPA), PEQ, and TAPES-Revised, to create a custom survey tailored to their research needs. Similarly, Brown et al. (2018) merged components from four existing questionnaires: Patient Assessment Validation Evaluation Test (PAVET), PEQ, Q-TFA, and TAPES. In addition to standard questionnaires, Gnyawali et al. (2023) investigated perceived sweat levels using a custom sweat score.

Mitton et al. (2017) used the SIGAM mobility score to assess amputee mobility during prosthesis fitting with different socket types. Kahle et al. (2016) applied the Pain Scale to identify various types of pain, hypothesizing that volume fluctuations negatively impact prosthesis control and comfort, potentially leading to pain and decreased usage. Specific questions targeted pain following amputation and prosthesis use. Aydın and Çağlar Okur (2018) used the Beck Depression Inventory (BDI) to analyze anxiety and depression among transfemoral amputees, finding no significant differences between those fitted with or without test sockets.

Additionally, five studies (5/25) incorporated individual, non-validated questionnaires, addressing preferences for socket shapes or wearing comfort (Hachisuka et al., 1999; Kahle and Highsmith, 2013; Henao et al., 2020; Kahle et al., 2021; Diment et al., 2022).

### 3.6 Comparative analysis of measurement methods

In the subsequent section, the analyzed studies were clustered based on their research focus on the socket-limb interface, including socket design, volume fluctuations, pressure/shear rates, pistoning, and other factors. This clustering enabled a targeted thematic comparison of the applied measurement methods and the derivation of trends. Table 4 provides an overview that correlates the partial findings described in the previous subchapters.

Five out of eleven studies (5/11) focusing primarily on socket design conducted mobility tests (Kahle and Highsmith, 2014; Brown et al., 2018; Fatone et al., 2021; Kahle et al., 2021; Pellegrini et al., 2021) or motion capture analyses (3/11) (Klotz et al., 2011; Brown et al., 2018; Pellegrini et al., 2021). Another commonly employed measurement method involves the use of pressure sensors (2/11) (Lee et al., 1997; Kahle and Highsmith, 2013) or force/pressure platforms (6/11) (Lee et al., 1997; Hachisuka et al., 1999; Kahle and Highsmith, 2014; Brown et al., 2018; Kahle et al., 2021; Pellegrini et al., 2021). In a total of five studies, X-ray imaging (4/11) (Hachisuka et al., 1999; Kahle and Highsmith, 2013; Kahle et al., 2020; Maikos et al., 2021) or fluoroscopy (1/11) (Brown et al., 2018) was utilized. Nine of the eleven “socket design” studies (9/11) supplemented their objective analyses with questionnaires, which included both validated and non-validated instruments as well as individualized questionnaires (Hachisuka et al., 1999; Kahle and Highsmith, 2013; 2014; Brown et al., 2018; Kahle et al., 2020; 2021; Fatone et al., 2021; Maikos et al., 2021; Pellegrini et al., 2021). The SCS was the most frequently utilized questionnaire across these studies (3/11) (Brown et al., 2018; Kahle et al., 2020; Fatone et al., 2021). Two studies developed questionnaires based on established validated tools, including the PAVET, PEQ, Q-TFA, and TAPES (Brown et al., 2018; Maikos et al., 2021).

The impact of residual limb volume fluctuations on the socket-limb interface has been examined in five studies (Kahle et al., 2016; Mitton et al., 2017; Paternò et al., 2021; Diment et al., 2022; Lee et al., 2023). Two of these studies utilized a 3D-scan system (2/5) to capture the surface geometry of the residual limb (Paternò et al., 2021; Diment et al., 2022). Additional measuring methods included mobility tests (Kahle et al., 2016), wearing time evaluations (Mitton et al., 2017), and pressure sensors (Lee et al., 2023). Subjective data were collected in three of the five studies (3/5) using questionnaires (Kahle et al., 2016; Mitton et al., 2017; Diment et al., 2022). The SCS was employed in two studies (2/5) (Kahle et al., 2016; Mitton et al., 2017). Moreover, a pain scale (Kahle et al., 2016) and a customized questionnaire (Diment et al., 2022) were utilized.

Four of the included studies investigated pressure and shear rates at the socket-limb interface (Henao et al., 2020; Gale et al., 2021; Tang et al., 2022; Paternò et al., 2024). Of these, two studies employed pressure and shear sensors (2/4) at the interface (Tang et al., 2022; Paternò et al., 2024). However, subjective measurements were not incorporated into either study. Additional methods for examining stump loading included DBR (Gale et al., 2021) and CT imaging, paired with FE analysis (Henao et al., 2020). These two studies were conducted in conjunction with the Q-TFA and a customized questionnaire, respectively.

Radiological examinations were utilized in all cluster-specific studies (3/3) investigating femur pistoning (Gale et al., 2020; Kahle et al., 2020; Maikos et al., 2021). Of these, two studies employed X-ray imaging (2/3) (Kahle et al., 2020; Maikos et al., 2021), while one study used DBR (Gale et al., 2020). Moreover, CT scans were conducted in two of the studies (2/3) (Gale et al., 2020; Maikos et al., 2021). All three objective measurements were paired with various questionnaires (3/3) (Gale et al., 2020; Kahle et al., 2020; Maikos et al., 2021). Participant responses were obtained using the Q-TFA, the SCS, and a combined questionnaire.

The utility of a test socket prior to definitive prosthetic fitting was evaluated in one study through mobility tests, alongside the TAPES questionnaire and the BDI (Aydın and Çağlar Okur, 2018). Furthermore, prosthesis alignment and misalignment were analyzed using a force platform and a thermal camera, without the inclusion of additional subjective assessments (Cárdenas et al., 2022). Perspiration was objectively measured using relative humidity and temperature sensors, combined with non-validated questionnaires, specifically the CLASS and a sweat score (Gnyawali et al., 2023).

## 4 Discussion

The reviewed literature underscores the complexity of challenges associated with the socket-limb interface for transfemoral amputees, emphasizing the critical need for optimized prosthetic designs and evaluation methods. Achieving optimal socket fit and design, managing residual limb volume fluctuations, and addressing pressure, shear stresses, and temperature are central to ensuring user comfort, functionality, and limb health. This review highlights significant heterogeneity in the methods and findings, providing insights into both objective and subjective approaches. Furthermore, the diversity of study designs and corresponding investigative methods reflects the complexity of the research question but also introduces variability that complicates comparisons and generalized conclusions.

### 4.1 Investigating socket design and fit

Socket design was the most frequently discussed topic among the reviewed studies, reflecting its central importance in optimizing prosthetic functionality. Displacements at the socket-limb interface, influenced by factors such as pressure, shear stresses, temperature, and volume changes, were identified as pivotal for user comfort and residual limb health (Paternò et al., 2018). Inadequate socket fit is closely linked to skin pathologies of the residual limb, highlighting the need for precise evaluation methodologies. Radiological techniques, gait analyses, and motion tests were commonly employed as objective approaches for evaluating socket design. Subjective assessments predominantly relied on validated tools like the SCS (3/11 studies) (Brown et al., 2018; Kahle et al., 2020; Fatone et al., 2021). However, methodological inconsistencies across studies hinder comparability, emphasizing the importance of universally validated questionnaires.

The choice of study design significantly impacts the reliability and applicability of findings. A notable example is the non-randomized study by Hachisuka et al. (1999), in which intervention groups tested only one socket design (IRC and QL). While this design provided insights into inter-group comparisons, it lacked the robustness of crossover trials. Crossover trials enable intra-subject comparisons under controlled conditions, reducing variability caused by individual differences in residual limb conditions. Consequently, they are considered superior for evaluating innovative socket designs. By minimizing external influences, these designs provide more reliable insights into user preferences, such as the favorability of brimless sockets for reducing pressure and enhancing comfort (Kahle and Highsmith, 2013).

Standardized questionnaires addressing daily function, comfort, and satisfaction would further enhance study comparability. Establishing an objective measurement method could not only streamline the iterative fitting process but also reduce potential biases introduced by subjective evaluations. Such integration is particularly important, given the diversity of methodologies observed in the reviewed literature.

### 4.2 Evaluating volume fluctuations

Residual limb volume fluctuations present a significant challenge due to the high proportion of soft tissue in transfemoral amputees (Paternò et al., 2021). Two studies employed 3D-scan systems to capture the residual limb’s surface geometry (Paternò et al., 2021; Diment et al., 2022). While these aligned methodologies suggest a promising approach, the inconsistency and limited number of studies restrict generalizable conclusions. Subjective evaluations employed tools like the SCS and customized questionnaires, or supplementary questionnaires such as the SIGAM mobility score and pain scales, which, while informative, lack validation and cross-study comparability (Kahle et al., 2016; Mitton et al., 2017; Diment et al., 2022).

Given the limited data, interpreting results regarding volume fluctuations is complicated. Methodological refinements and a broader evidence base are required to comprehensively assess the influence of volume changes on prosthetic performance and to validate emerging measurement methods. The lack of standardization and the low number of studies highlight gaps in the evidence necessary for generalized conclusions.

### 4.3 Pressure and shear stress analysis

Pressure and shear stresses, closely linked to skin pathologies of the residual limb, were investigated using varied approaches. Only half of the relevant studies employed pressure and shear sensors (2/4) (Tang et al., 2022; Paternò et al., 2024). Early studies, such as those by Lee et al. (1997), used pressure transducers embedded in the socket, but their potential to influence measurements was noted, too. Recent advancements, including ultra-thin sensors (<0.2–1 mm), aimed to mitigate this issue (Tang et al., 2022; Paternò et al., 2024). Despite these improvements, feedback-dependent sensor placement introduces subjectivity, highlighting the need for contactless or fully integrated measurement techniques.

Additionally, Gale et al. (2021) identified a negative correlation between shear rates on the residual limb and prosthesis usage, suggesting that high shear rates adversely affect comfort and adherence. The data from Gale et al. (2020) reinforced this connection by correlating increased femoral pistoning with deteriorated Q-TFA scores. Henao et al. (2020) suggested pressure-tolerant and pressure-sensitive areas on the residual limb, based on a combination of objective and subjective measurements. However, the limited number of studies and methodological inconsistencies in this area preclude the establishment of standardized protocols. Improved sensor integration and expanded studies are essential for refining pressure and shear stress evaluations.

### 4.4 Insights into femur pistoning

Femur pistoning, a critical aspect of socket-limb interface evaluation, was predominantly examined using radiological techniques, such as X-ray imaging, CT scans, and dynamic stereo radiography (Gale et al., 2020; Kahle et al., 2020; Maikos et al., 2021). CT scans enabled the generation of 3D bone models, while X-ray imaging provided insights into socket brim interactions and femur movement. Despite these advancements, inconsistencies in methodologies and limited data availability hamper broader conclusions. Gale et al. (2020) noted correlations between increased pistoning and deteriorated Q-TFA scores.

Expanding the scope of studies and standardizing imaging protocols could facilitate more robust evaluations of pistoning dynamics and their impact on comfort and functionality.

### 4.5 Advancing research and prosthetic design

Few studies have integrated objective measurements with subjective questionnaire data, underscoring a significant gap in comprehensive evaluation frameworks. For instance, Kahle and Highsmith (2013) demonstrated that the brimless IRC VAS design reduced pressure in proximal medial regions (190 mmHg compared to 322 mmHg in the standard design), with participants favoring the brimless socket for enhanced comfort. Similarly, Kahle et al. (2021) observed improved gait symmetry parameters in users of DS and Sub-I sockets compared to IRC sockets. A total of 92% of participants preferred one of these designs (46% preferred DS, 46% preferred Sub-I sockets), primarily due to enhanced comfort, stability or ROM.

Such findings emphasize the interplay between physical parameters, such as pressure distribution and shear rates, and subjective satisfaction. Standardized approaches that combine validated questionnaires with advanced technological solutions are essential to refine socket design and evaluation methods. Furthermore, the limited number of studies on topics such as test sockets, prosthesis alignment, and perspiration highlights the need for broader investigations to support more generalized conclusions.

### 4.6 Limitations

This systematic review has several limitations. Firstly, it is possible that not all relevant publications were identified or included, despite a comprehensive search strategy across multiple databases. Additionally, the included literature comprises, among others, seven case studies with a low LoE. Nonetheless, these studies were incorporated due to their methodological contributions, as the primary focus of this review concerns the evaluation of measurement methods rather than specific study outcomes.

In order to comprehensively cover the variety of available measurement methods, no restrictions were applied during the screening process. However, this approach resulted in the inclusion of non-validated measurement methods, particularly questionnaires, which further complicates the comparability of the study results.

The lack of a standardized acclimation period for socket studies, as noted by Kahle et al. (2021), represents another limitation. Varying acclimation periods across the included studies reduce the comparability of subjective and objective outcomes, potentially introducing variability in the assessment of socket fit and comfort. Additionally, the use of questionnaires as subjective measurement tools inherently relies on self-assessment, which may lead to biases such as over- or under-reporting, thus limiting the objectivity and reproducibility of findings.

Furthermore, this review did not impose restrictions on the cause of amputation to ensure a comprehensive analysis of the examined measurement methods. However, it should be noted that amputations resulting from diabetes and/or vascular disease are often associated with patient groups that have a higher prevalence of comorbidities and an older age profile. Consequently, these patients generally have lower rehabilitation prospects compared to younger amputees with traumatic lower limb loss (Moore et al., 1989). As a result, the generalizability of certain findings may be limited.

The influence of additional prosthetic components, such as liners and suspensions, represents another limitation of this review. Although the socket-limb interface remains the primary focus, these components interact dynamically with the socket and significantly affect overall fit and comfort, which underscores the need to account for them in evaluations. For instance, while the study by Cárdenas et al. (2022) excluded liners, providing a distinct perspective, most of the included studies utilized varied combinations of liners and suspensions, likely influencing the findings.

Lastly, inconsistencies in the methodologies of objective measurement tools across studies, including pressure sensor calibration and motion analysis systems, underline the need for standardized protocols. These inconsistencies further limit the comparability of findings and reinforce the necessity of developing unified frameworks for evaluating socket fit and comfort.

### 4.7 Conclusion

As the socket-limb interface is critical, this systematic review emphasizes the importance of evaluating socket fit and comfort in transfemoral amputees using both subjective and objective methods. The findings reveal significant gaps in current approaches, particularly the absence of a suitable validated, standardized objective measurement method for assessing socket fit and comfort.

Validated subjective questionnaires, including tools like the SCS and PEQ, provide valuable insights into patient-reported outcomes. However, their reliance on self-reporting limits their objectivity and comparability across studies. Objective measurement methods, such as pressure sensors, motion analyses, and dynamic radiographic imaging, demonstrate potential for enhancing precision and reducing biases in socket evaluations. Nonetheless, methodological inconsistencies and the lack of standardized protocols restrict their broader application.

The results of this review underline the urgent need for a standardized approach that integrates the strengths of objective tools and validated subjective questionnaires. Such framework would enable reliable comparisons across studies, drive innovations in socket design, and enhance patient satisfaction and prosthesis functionality.

Relating to the PICO framework, this review aimed to assess whether technical analyses of the socket-limb interface enable a more reliable determination of prosthetic fit and comfort for unilateral transfemoral amputees compared to subjective evaluations. The findings indicate that neither objective methods nor subjective tools alone are sufficient to address the multifaceted challenges of the socket-limb interface. Instead, a combined approach leveraging quantitative data and patient-reported outcomes is essential for developing robust and personalized solutions. Addressing the methodological gaps identified in this review will allow future research to refine objective measurement methods and reliably evaluate socket fit and comfort. These advancements are expected to provide the foundation for developing adaptive, personalized socket designs informed by precise, validated data, better accommodating individual physiological needs and optimizing prosthetic performance.

## Data Availability

The original contributions presented in the study are included in the article/Supplementary Material, further inquiries can be directed to the corresponding author.

## Glossary

2MWT: 2-Minute Walk Test
4SST: 4-Square-Step-Test
5T Rapid STST: 5-Times Rapid Sit-to-Stand Test
6MWT: 6-Minute Walking Test
AMP: Amputee Mobility Predictor
BDI: Beck Depression Inventory
CAT-CAM: Contoured Adducted Trochanteric-Controlled Alignment Method
CLASS: Comprehensive Lower Limb Amputee Socket Survey
CRPS: Complex Regional Pain Syndrome
CRS: Compression/Release Stabilization
CT: Computer Tomography
DBR: Dynamic Biplane Radiography
DS: Dynamic Socket
ECG: Electrocardiogram
EMG: Electromyography
HySS: Hybrid Subischial Socket
IC: Ischial Containment
IMU: Inertial Measurement Units
IRC: Ischial Ramus Containment
LA: Language
LoE: Level of Evidence
MA: Marlo Anatomical
NU-FlexSIV: Northwestern University-Flexible Subischial Vacuum
OPUS: Orthotic and Prosthetic Users’ Survey
PAVET: Patient Assessment Validation Evaluation Test
PCI: Physiological Cost Index
PEQ: Prosthesis Evaluation Questionnaire
PICO: Participant, Intervention, Comparison, and Outcome
PPA: Prosthetic Profile of the Amputee
PRISMA: Preferred Reporting Items for Systematic Reviews and Meta-Analyses
PROSPERO: International Prospective Register of Systematic Reviews
PVD: Peripheral Vascular Disease
ROM: Range of Motion
QL: Quadrilateral
Q-TFA: Questionnaire for Persons with a Transfemoral Amputation
SATPRO: Satisfaction with Prosthesis
SCS: Socket Comfort Score
Sub-I: Sub-Ischial
TAPES: Trinity Amputation and Prosthetic Experience Scales
THR: Total Hip Replacement
TUG: Timed Up and Go Test
VAS: Vacuum-Assisted Suspension
