# Supplementary material for: Objective measurement methods for the evaluation of socket comfort in patients with transfemoral amputation: a systematic review
Source: Front Bioeng Biotechnol. 2025 May 30;13:1576729. doi: 10.3389/fbioe.2025.1576729 (PMC12163241; doi:10.3389/fbioe.2025.1576729)
Supplement: Supplementary file 1 [file DataSheet1.docx]

Supplementary Material

**Methodological Quality Criterion** (van der Linde et al., 2004; Brodie et al., 2022)

**Selection of Patients – A criteria**

- A1 – Adequacy of Description of Inclusion and Exclusion Criteria: This criterion tested whether the participant sample was sufficiently defined. If at least four of the following points are mentioned, this criterion was scored “1”:
  - Age, Gender, Level of amputation, Reason for amputation, Activity level of participant, Time since onset, Residuum condition (tissue consistency, length of stump, scar), Comorbidity
- A2 – Functional Homogeneity: The homogeneity of the study sample was assessed for all study designs. In view of clinical guideline development, at least the activity level of the included subjects should be reasonably equal (K-Level). When this is not described, sufficient indication of amputation level, reason for amputation, and age of the participants were required to globally estimate the activity level. If the study sample was heterogeneous, a stratified analysis of the outcome was needed to obtain a “1” score, if the study sample consisted of only one participant the score was “0” because no homogeneity of several participants could be reached
- A3 – Prognostic Comparability: As for group designs, the study groups should be comparable for possible confounding factors (time since onset and time since first walking with the prosthesis, accommodation time, same prosthetist). In the case of a within-subjects design, this criterion was scored “1”, if the daily used prosthesis was compared to a new fitted prosthesis the score was “0” because different prosthetists manufactured the sockets, if the study included only one subject, the score was rated with “0”
- A4 – Randomization: An adequate randomization procedure should have been applied in group designs. If this procedure was described and reasonably excluded bias, this criterion was scored as “1.” In within-subject designs, this criterion was applied to the sequence of interventions (Piantadosi, 1997)

**Intervention and Assessment – B criteria**

- B5 – Experimental Intervention: The experimental intervention had to be given explicitly in such detail to allow a duplicate study to be performed
- B6 – Cointerventions: This criterion tested whether cointerventions were avoided or were comparable between the study groups (additional procedure)
- B7 – Blinding: The assessor should be blinded to the intervention. If this was ensured in the study, a score of “1” was recorded. When investigating prosthetic components, blinding of the patients is always difficult to ensure. In our review, no study could ensure this, so we focused on the blinding of assessors.
- B8 – Timing of the Measurement: This criterion pertained to the moment that the outcome was assessed in relation to the time period subjects were given to adapt to the prosthetic change. For this review, there was no adequate acclimation period for transfemoral socket designs; therefore, all studies attained “1”
- B9 – Outcome Measures: The outcome parameters should be adequate in relation to the purpose of the study, and they should have been collected with the use of a standardized protocol

**Statistical Validity – C criteria**

- C10 – Dropouts: The number of dropouts and the reason for dropping out had to be sufficiently reported. This criterion was scored “1” if no dropouts occurred or if they had been sufficiently reported. However, a dropout rate of more than 20% was considered insufficient and therefore scored “0”
- C11 – Sample Size: The sample size (n) in relation to the number of independent variables (K) was adequate if the ratio n:K exceeded 10:1. For this review – the number of participants completed the protocol:the intervention (e.g. different sockets, with or without test socket, suspension)
- C12 – Data Presentation: This criterion required that adequate point estimates and measures of variability were presented for the primary outcome measures

Piantadosi S. Clinical trials as experimental designs. Clinical Trials. A Methodological Perspective. 1997.
